# N-3 (*Omega*-3) Fatty Acids in Postpartum Depression: Implications for Prevention and Treatment

**DOI:** 10.1155/2011/467349

**Published:** 2010-10-27

**Authors:** Beth Levant

**Affiliations:** Department of Pharmacology, Toxicology, and Therapeutics, Kansas Intellectual and Developmental Disabilities Research Center, The University of Kansas Medical Center, MS-1018, 3901 Rainbow Boulevard., Kansas City, KS 66160, USA

## Abstract

A growing body of clinical and epidemiological evidence suggests that low dietary intake and/or tissue levels of n-3 (*omega*-3) polyunsaturated fatty acids (PUFAs) are associated with postpartum depression. Low tissue levels of n-3 PUFAs, particularly docosahexaenoic acid (DHA), are reported in patients with either postpartum or nonpuerperal depression. Moreover, the physiological demands of pregnancy and lactation put childbearing women at particular risk of experiencing a loss of DHA from tissues including the brain, especially in individuals with inadequate dietary n-3 PUFA intake or suboptimal metabolic capabilities. Animal studies indicate that decreased brain DHA in postpartum females leads to several depression-associated neurobiological changes including decreased hippocampal brain-derived neurotrophic factor and augmented hypothalamic-pituitary-adrenal responses to stress. Taken together, these findings support a role for decreased brain n-3 PUFAs in the multifactorial etiology of depression, particularly postpartum depression. These findings, and their implications for research and clinical practice, are discussed.

## 1. Introduction

Postpartum depression is a potentially devastating disorder that occurs in 10%–20% of childbearing women [1–3]. The etiology remains to be fully elucidated; however, it is complex, most likely heterogeneous, and probably involves the interaction of environmental factors and genetic predispositions, with pregnancy or childbirth as the triggering event [4–10]. Hormonal changes during pregnancy and after childbirth appear to play a contributory role, but cannot fully explain incidence of the disorder [11–13]. Untreated, postpartum depression can lead to recurrent depressive episodes, negatively affect the development of the infant, and in severe instances, lead to maternal suicide or infanticide [14–16]. There is thus a critical need to elucidate causes and risk factors of this disorder that affects the health and well-being of both mothers and infants in order to identify means of prevention or treatment. A growing body of evidence suggests that n-3 polyunsaturated fatty acid (PUFA) status may contribute to the development of postpartum depression. That literature, and its implications for research and clinical practice, is reviewed here.

## 2. Methods

This paper is based on literature found in PubMed searched from 1964 through July 31, 2010. Primary outcomes of interest were the effects of pregnancy and lactation on maternal n-3 PUFA status in humans and in animals, relationships between n-3 PUFA status and postpartum depression, as well as clinical trials of n-3 PUFAs in postpartum depression. Secondary clinical outcomes of interest were relationships between n-3 PUFA status and non-puerperal depression, and clinical trials of n-3 PUFAs in non-puerperal depression. Preclinical outcomes of interest were the effects of manipulations of dietary or tissue n-3 PUFA status on neurobiological systems known to be altered in depression (i.e., hippocampal expression of brain-derived neurotrophic factor, the hypothalamic-pituitary-adrenal axis, the CNS serotonin and dopamine systems, and neuroinflammation). Effects of altered n-3 PUFA status in animal models of depression were also examined.

## 3. N-3 Polyunsaturated Fatty Acids

**Figure 1:**
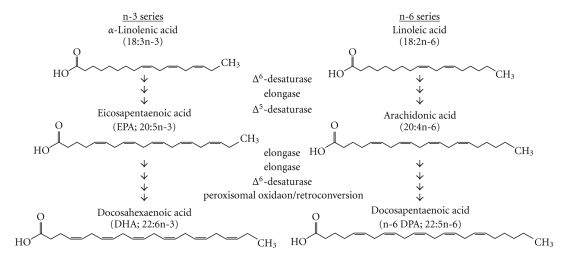
Biosynthesis of n-3 and n-6 polyunsaturated fatty acids. The essential fatty acids *α*-linolenic acid and linoleic acid are metabolized by elongases and desaturases into a variety of n-3 and n-6 LC-PUFA, respectively.

Long-chain polyunsaturated fatty acids (LC-PUFAs) are fatty acids that are 20 or more carbons in length and contain multiple double bonds. The n-3 and n-6 (or *omega*-3 and *omega*-6) families of PUFAs are synthesized from the nutritionally essential fatty acids, *α*-linolenic acid (18:3n-3; which indicates the number of carbons: the number of double bonds, and the fatty acid family) and linoleic acid (18:2n-6) (Figure 1). Biologically important LC-PUFAs such as docosahexaenoic acid (DHA; 22:6n-3) and arachidonic acid (20:4n-6) can either be synthesized from the essential fatty acids or consumed directly in the diet from sources such as fatty fish, which is notably rich in n-3 LC-PUFAs, and other animal products. However, humans are relatively inefficient in synthesizing LC-PUFAs (≤6% conversion) from the essential fatty acids [17, 18], which may be further exacerbated by genetic polymorphisms that render certain individuals particularly poor at synthesizing or utilizing LC-PUFAs [19, 20]. Compounding this metabolic inefficiency, diets in North America are notably low in n-3 PUFAs, particularly relative to n-6 PUFAs, which compete for metabolism into LC-PUFAs [21]. Thus, there is considerable potential for variation in n-3 PUFA status between populations and between individuals within a given population.

LC-PUFAs are components of the phospholipids that form cell membranes. The phospholipids in brain have notably high concentrations of LC-PUFAs, with DHA being the most abundant species. Variation in fatty acid composition of the phospholipids alters the physicochemical properties of the membrane and can thus alter the function of membrane-bound proteins and lipid rafts [22, 23]. DHA and other LC-PUFAs can also be cleaved from the membrane by phospholipases to serve as precursors for inter- and intracellular signaling molecules such as prostaglandins, neuroprotectin D1, and resolvins [22, 24–26]. In addition, LC-PUFAs are agonists at nuclear receptors, such as the retinoid X receptor (RXR) and peroxisome proliferator-activated receptors (PPAR), which modulate gene expression [27, 28]. Thus, changes in the relative abundance of specific LC-PUFAs, particularly DHA, can affect neuronal function through a variety of mechanisms. 

Most of the DHA in the human brain accumulates during the third trimester of gestation, and continues through the first few years of life [29–31]. DHA is supplied by the mother to the fetus *in utero,* and to the neonate in breast milk which contains high concentrations of DHA, though concentrations vary depending on maternal diet and other factors [32, 33]. Low availability of DHA results in increased incorporation of docosapentaenoic acid (n-6 DPA, 22:5n-6), the 22-carbon member of the n-6 PUFA family, thus altering the fatty acid composition of the phospholipids [34]. This change in the composition of brain phospholipids does not affect brain weight or overall growth [35] but is associated with suboptimal visual, attentional, and intellectual development [36–39].

## 4. Effects of Pregnancy and Lactation on Maternal N-3 PUFA Status

**Figure 2:**
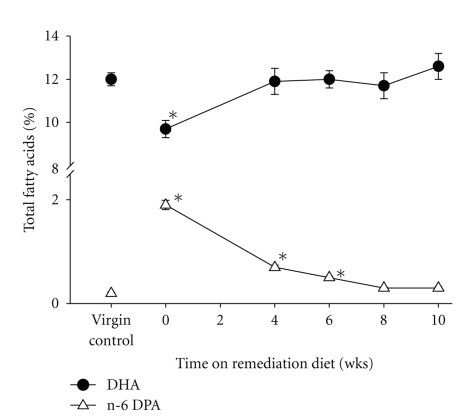
Effects of dietary remediation on brain phospholipid DHA and n-6 DPA contents in postpartum dams with a reproduction- and diet-related decrease in brain DHA. Adult female rats underwent two complete reproductive cycles (pregnancy and lactation) while fed an n-3 PUFA-deficient diet as previously described [49]. At the time of weaning of the second litter, dams were placed on a remediation diet containing DHA (4% of fat by weight). Virgin controls were age-matched females fed a control diet for 12 weeks. *n* = 6/group. Data are presented as the mean ± SEM. *P < .05 versus virgin control by ANOVA and Tukey's test.

As the source of nutrition for the developing fetus and infant, there is considerable demand on pregnant and nursing women to supply DHA to their offspring [37, 38]. Without an adequate diet, mothers can become depleted of nutrients. The majority of studies reported that maternal plasma DHA levels were decreased by as much as 50% in some individuals after a single pregnancy, and were not fully replenished at 26 weeks postpartum [40–44]. Additional pregnancies resulted in further reduction of maternal DHA levels in plasma and breast milk [43, 45]. Similar decreases in the percentage of DHA in erythrocytes and liver have also been reported after pregnancy and lactation in rats [46]. Although brain fatty acid status has not been studied in humans after pregnancy, studies in rats indicated that the DHA content of brain phospholipids was reduced by roughly 25% after only a single reproductive cycle (i.e., pregnancy and lactation through weaning) if the animals were fed a diet low in n-3 PUFAs [47, 48]. The percentage of DHA in rat brain was not further decreased after multiple reproductive cycles; however, a second reproductive cycle resulted in additional incorporation of n-6 DPA [48]. While these reproduction-associated changes in brain fatty acid composition can be reversed by subsequent treatment with DHA (Figure 2), it is not yet known whether this restoration of brain fatty acid composition reverses the neurobiological changes that result from the loss of DHA (see below).

## 5. N-3 PUFAs in Depression

### 5.1. Postpartum Depression

Epidemiologic and clinical studies suggest that pregnancy-associated changes in n-3 LC-PUFA status contribute to the development of postpartum depression. A cross-national analysis indicated that higher fish consumption, which was reflected in higher concentrations of DHA in breast milk, correlated with a lower incidence of postpartum depression [50]. Low intake of fish and other sources of n-3 PUFAs was also associated with depression during pregnancy [51, 52]. Brain fatty acid composition in postpartum depression has not been studied. Nonetheless, in studies of plasma or serum, DHA concentrations, or the DHA:n-6 DPA ratio, was significantly lower in postpartum women experiencing depressive symptoms than those who were not [53, 54]. Similarly, women who later developed postpartum depression had lower serum DHA levels after delivery than those who did not develop depressive symptoms [55], although other studies did not find such a relationship [56–58]. Likewise, risk of postpartum depression was associated with a single-nucleotide polymorphism in the FADS1/FADS2 gene cluster [59], which encodes the rate-limiting enzymes in LC-PUFA biosynthesis, and was associated with lower proportions of DHA in breast milk even if the women were consuming fish or fish oil [60]. In addition, women with more than one child or who had short interpregnancy intervals (<24 months) were found to be at higher risk of developing postpartum depression [9, 61], consistent with the potential for greater alterations in n-3 LC-PUFA status after multiple pregnancies and/or inadequate time for replenishment between pregnancies.

### 5.2. Nonpuerperal Depression

 Low n-3 PUFA status, particularly low DHA, has also been reported in non-puerperal depression. Analyses of dietary n-3 PUFA intake indicate an association of low n-3 PUFA consumption with depressive symptoms [62–66]. Likewise, in postmortem studies, tissue DHA content of the orbitofrontal cortex was decreased 22% in individuals with major depressive disorder compared to controls and was the only fatty acid found to be altered [67]. Similarly, the DHA content of the cingulate cortex was lower in individuals with major depression, but was one of several fatty acids found to be altered [68]. Studies of erythrocyte, serum, plasma, or adipose tissue levels of DHA and other n-3 PUFAs have found similar results [69–79]; findings that have been supported by subsequent meta-analysis [80]. Interestingly, some studies found stronger associations of low n-3 PUFA status in women than in men, or found an association only in women [67, 81, 82], in whom depression occurs with roughly twice the frequency as men [83–85]. Expression of FADS1 and several other genes involved in lipid metabolism was also decreased in individuals who completed suicide [86]; however, no alterations in any of the n-3 PUFAs were found in postmortem brains from suicide completers, even in those individuals with a diagnosis of major depression [87, 88].

## 6. Clinical Trials with N-3 PUFAs in Depression

Clinical trials with n-3 PUFAs in depression have yielded varying results. These differences in outcomes are likely due to the considerable variation in the n-3 PUFA preparations used, as well as numerous other differences between the studies including the dose, duration of treatment, severity of the depressive symptoms, and inclusion/exclusion criteria, percentage of male and female subjects, choice of placebo, and the inclusion of other concomitant treatments such as psychotherapy. In addition, the populations studied in these clinical trials also varied in their dietary n-3 and n-6 PUFA content, which may also have contributed to the variable outcomes. Moreover, several of the studies likely lacked adequate statistical power. 

### 6.1. Postpartum Depression

Only a few studies have examined the effects of n-3 PUFA treatment specifically in postpartum depression. Treatment with a preparation containing DHA and eicosapentaenoic acid (EPA, 20:5n-3) (EPAX 550 [EPA : DHA, 1.5 : 1] 0.5, 1.4, or 2.8 g/day) for 8 weeks reduced depressive symptoms in postpartum depression in a dose-ranging pilot study that did not include a placebo control group [89]. In a double-blind, placebo-controlled trial, however, treatment with DHA and EPA (0.8 and 1.1 g/day, resp.) for 8 weeks was of no additional benefit in women with perinatal major depression when all subjects received concomitant psychotherapy [90]. Fish oil (2960 mg/day [EPA : DPA, 1.4 : 1] from week 34–36 of pregnancy through 12 weeks postpartum) [91] or DHA supplements (200 mg/day for 4 months after delivery [92] or 220 mg/day from week 16 of pregnancy through 3 months postpartum [93]) also failed to prevent the development of postpartum depressive symptoms [91–93].

### 6.2. Nonpuerperal Depression

The effects of n-3 PUFAs in non-puerperal depression have been more extensively tested (Table 1). Double-blind, placebo-controlled clinical trials in depressed patients found that various n-3 LC-PUFA preparations, such as EPA, a combination of EPA and DHA, or fish oil, were beneficial as an adjunct to the patients' current antidepressant medication [94–96] or when administered as monotherapy [97–100], for at least some doses or in certain subsets of subjects. Similarly, fish oil improved depressive symptoms in depressed Parkinson's patients [101]. In other studies that did not include a placebo control, ethyl-EPA had equal efficacy to fluoxetine, and the combination of ethyl-EPA and fluoxetine produced greater improvement than fluoxetine alone [102]. Some doses of DHA alone also improved depressive symptoms in a dose-ranging study [103]. On the other hand, other double-blind, controlled clinical trials using DHA alone, combinations of DHA and EPA, or fish oil found no antidepressant effects [104–106], and EPA produced no beneficial effects alone or as an add-on to antidepressant medication [107–109]. Despite the negative results of some trials, subsequent meta-analyses and other post hoc evaluations generally support the antidepressant efficacy of n-3 PUFAs, particularly EPA, though the efficacy of DHA remains unclear [110–114]. These analyses also highlight the need for additional controlled randomized trials with larger sample sizes and adequate doses and treatment durations.

## 7. Biological Mechanisms for N-3 PUFAs in Postpartum Depression

Experimental studies in animals and correlational studies in humans indicate several biological mechanisms by which variation in n-3 PUFA consumption and/or tissue n-3 PUFA status may contribute to the pathogenesis of depression. The vast majority of animal studies have used diets to modulate the availability of specific LC-PUFAs, and thus, tissue fatty acid compositions. On the other hand, altered brain LC-PUFAs in humans may result from genetic variation in PUFA metabolism or utilization, perhaps also in combination with inadequate diet. Nevertheless, the effects of altered brain LC-PUFA composition should likely be similar regardless of the underlying cause. However, the neurobiological consequences of variation in n-3 PUFA status do vary depending on the magnitude of the change, the point in the lifespan at which the manipulation occurred, and in some instances, the physiological state (e.g., postpartum). Accordingly, the effects of variation in brain n-3 PUFA status on neurobiological parameters known to be of importance in depression are reviewed here with a focus on the effects in postpartum females.

### 7.1. Effects on Hippocampal Expression of Brain-Derived Neurotrophic Factor (BDNF)

 Decreased expression of BDNF in the hippocampus, a component of the limbic system involved in memory, affect, and regulation of the hypothalamic-pituitary-adrenal axis [115], is strongly implicated in the pathophysiology of depression. Of note, hippocampal BDNF levels were decreased in suicide completers [116, 117]. This decrease in BDNF expression results in decreased hippocampal neurogenesis [118], and may consequently contribute to the hippocampal atrophy observed in depression [119]. Furthermore, BDNF levels were higher in postmortem hippocampus of antidepressant-treated patients than in untreated patients, suggesting a role for BDNF in the mechanism of antidepressants [120]. Similar effects have been observed in animal studies with various models of depression, stress paradigms, and antidepressant treatments [121–127].

When examined at the time of weaning a litter, which is at the end of the period of greatest offspring demand for DHA in rodents [128], and thus roughly comparable to the postpartum period in humans, female rats that experienced a decrease in brain DHA as a result of pregnancy and lactation while being fed an n-3 PUFA-deficient diet exhibited decreased hippocampal BDNF mRNA and peptide levels [49]. These animals had a decrease in brain DHA content of about 25% which is similar to the decrease observed in postmortem brain samples from individuals with major depressive disorder [67]. Furthermore, the magnitude of the decrease in BDNF mRNA (−32%) was similar to that observed in suicide victims [116, 117]. A decrease in hippocampal BDNF mRNA levels was also observed in virgin female rats that were fed an n-3 PUFA-deficient diet for a sufficient period of time (6 months) to decrease brain DHA content by about 25% [49]. These effects on BDNF could not be attributed to differences in general health, weight gain, maternal offspring burden, or serum estradiol levels [49, 129]. This suggests that the decrease in hippocampal BDNF expression is related to brain DHA status specifically, not an interaction of brain DHA level and reproductive status. Even so, this effect was somewhat greater in parous females [49], suggesting that there may be some augmentation in the postpartum state.

Findings in other animal models and in humans also indicate a role for n-3 PUFAs in the regulation of hippocampal BDNF expression and function. Notably, a DHA-enriched diet in rats increased hippocampal expression of BDNF and also increased concentrations of molecules involved in BDNF signaling such as calmodulin kinase II and activated Akt [130]. Similarly, an increase in hippocampal BDNF was observed in adult mice treated with *α*-linoleic acid injections [131]. Consistent with this observation, mice or rats fed diets enriched in n-3 PUFAs had increased expression of hippocampal BDNF and either increased hippocampal neurogenesis or hippocampal volume [132, 133]. Likewise, in humans, higher consumption of n-3 LC-PUFAs was associated with increased gray matter volume in hippocampus and other corticolimbic structures, indicating maintenance of cells in those brain regions [134]. Thus, these data suggest that n-3 PUFAs support expression of hippocampal BDNF, which in turn fosters optimal hippocampal function.

### 7.2. Effects on the Hypothalamic-Pituitary-Adrenal Axis

 Dysregulation of the hypothalamic-pituitary-adrenal axis is another major clinical finding in depression [135]. These findings include elevated basal levels of serum cortisol, increased corticotrophin-releasing factor in cerebral spinal fluid [136–138], and disruption of negative feedback mechanisms [139]. 

In postpartum rats with decreased brain DHA levels, stress-induced corticosterone secretion was higher than in postpartum rats with normal brain DHA levels [49]. In addition, both postpartum and virgin female rats with decreased brain DHA exhibited greater relative increases in corticosterone secretion over baseline when subjected to an intense stressor [49], although stressed corticosterone levels were not different between virgin females with decreased brain DHA and virgin females with normal brain DHA. This suggests that a loss of DHA from the adult brain contributes to dysregulation of the hypothalamic-pituitary-adrenal axis and that this effect may be more pronounced in the postpartum state. 

Consistent with these findings, other animal and clinical studies support a role for dietary and tissue n-3 PUFA status in the modulation of the hypothalamic-pituitary-adrenal axis. In rats, an n-3 PUFA-enriched diet resulted in lower levels of anxiety- and stress-like behavioral effects in the elevated plus maze and the open field test after treatment with interleukin-1, an inflammatory cytokine that increases corticosterone levels [140]. Similarly, in human studies, fish oil supplements decreased stress responses such as increased plasma epinephrine, norepinephrine, and cortisol, in normal subjects [141, 142]. Furthermore, in a study of perpetrators of domestic violence, DHA levels were inversely related to concentrations of corticotropin-releasing factor in cerebrospinal fluid [143]. Thus, dietary and tissue n-3 PUFA levels appear to modulate the function of the hypothalamic-pituitary-adrenal axis in both the non-puerperal and postpartum states, and the effects of lower n-3 PUFAs are similar to the alterations observed in depressed patients.

### 7.3. Effects on the CNS Serotonin Systems

Decreased serotonergic function plays a central role in the theories of the pathogenesis of depression. This is supported by observations such as decreased concentrations of serotonin in the brainstem and increased densities of serotonin receptors, such as 5-HT_1A_ and 5-HT_2A_, in the prefrontal cortex of postmortem depressives and suicide victims [144–150]. These receptors downregulate after treatment with antidepressant drugs which increase synaptic availability of serotonin [151]. 

Postpartum rats with decreased brain DHA levels had increased expression of 5-HT_1A_ receptors in the hippocampus [49]. No alterations in hippocampal 5-HT_1A_ binding were observed in virgin females with decreased brain DHA indicating that this represents an interaction of decreased brain DHA content with the postpartum state. However, this observation differs from findings in depressed humans in whom densities of hippocampal 5-HT_1A_ receptors were either not altered or were decreased [152–155]. Furthermore, the densities of 5-HT_1A_ and 5-HT_2A_ receptors were not altered in the frontal cortex of either postpartum or virgin female rats with decreased brain DHA [49]. Thus, these findings are inconsistent with findings in humans with non-puerperal depression. Nevertheless, the increase in hippocampal 5-HT_1A_ receptors may represent a unique effect of a loss of brain DHA in postpartum dams that may contribute specifically to the yet-to-be determined etiology of postpartum depression. 

Other studies in animals and humans indicate that various aspects of the serotonin system are affected by n-3 PUFA status. In animal studies, adult female rats with a diet-induced decrease in brain DHA content of about 25% initiated after adulthood had decreased concentrations of serotonin in the frontal cortex [49]. Similarly, rats fed an n-3 PUFA-deficient diet from birth, which produced brain DHA levels 61% lower than controls as a result of inadequate accumulation during postnatal development, exhibited decreased midbrain expression of tryptophan hydroxylase, the enzyme that synthesizes serotonin, and increased serotonin turnover in the prefrontal cortex [156]. Consistent with these findings, piglets fed formula lacking both *α*-linolenic and linoleic acids exhibited lower cortical serotonin concentrations than those fed a formula containing the essential fatty acids [157], further suggesting a role for brain LC-PUFA composition in modulating serotonin levels though the specific role of n-3 PUFAs was not addressed. In another model, rats raised for two generations on an n-3 PUFA-deficient diet, which resulted in a 75% decrease in brain DHA content, had increased density of 5-HT_2A_ receptors in the frontal cortex [158, 159]. Conversely, an n-3 PUFA-supplemented diet reversed decreases in brain serotonin levels in mice subjected to unpredictable chronic mild stress [160]. In humans, low plasma DHA levels in normal subjects and alcoholics were correlated with lower concentrations of the serotonin metabolite 5-hydroxyindoleacetic acid in cerebrospinal fluid, a marker of altered serotonergic neurotransmission associated with depression and suicide [161, 162]. Similarly, the density of platelet serotonin transporter binding, another marker of depression and suicide, was also correlated with plasma DHA levels [163]. Thus, many of the serotonergic alterations associated with low dietary or tissue n-3 PUFAs are consistent with those observed in depression.

### 7.4. Effects on the CNS Dopamine Systems

Although the monoamine theory of depression focuses on serotonin and norepinephrine [164], the CNS dopamine systems also appear to play a role in the disease. Decreased dopaminergic function, particularly of the mesolimbic system, appears to underlie anhedonic behavior in several animal models [165–168]. Notably, concentrations of homovanillic acid, a dopamine metabolite, in cerebrospinal fluid were decreased in depressed patients and in suicide victims, and were inversely related to depression scores [169–172]. Depression is also common in Parkinson's disease, a neurodegenerative disease involving the loss of nigrostriatal dopamine neurons [173, 174]. Accordingly, decreased dopaminergic function has been hypothesized to contribute to the anhedonia and motivational deficits associated with depression [175].

Postpartum rats with decreased brain DHA levels exhibited decreased density of D_2_-like dopamine receptors in the ventral striatum (nucleus accumbens and olfactory tubercle) [129]. A trend towards a decrease in D_2_-like receptor binding was also observed in virgin females with decreased brain DHA, suggesting that the decrease in D_2_-like receptor binding in this brain region resulted from the change in brain DHA status, but may be augmented in the postpartum female. While this observation is consistent with the proposed hypoactivity of the mesolimbic dopamine system in depression, a postmortem study of drug-naïve patients with major depressive disorder found no differences in the density of D_2_ receptors in either the ventral striatum or the caudate nucleus [176]. Nevertheless, decreased densities of D_2_-like receptors or D_2_ receptor mRNA in the nucleus accumbens have been reported in several putative rat models of depression including chronic mild stress-induced anhedonia, the socially isolated Flinders sensitive line rat, and the learned helplessness model [177–179]. Decreased density of D_2_-like receptors was also observed in the nucleus accumbens core of the Wistar-Kyoto rat, another depression model, though D_2_-like receptor binding was increased in the nucleus accumbens shell [180]. 

Variation in diet and tissue n-3 PUFA content in other animals models also results in alterations in the CNS dopamine systems, but these effects vary considerably depending on the magnitude of the change and the point in development when the manipulation was made. For example, in contrast to the effects of a loss of brain DHA in adult animals, either an increase or no change in the density of D_2_ receptors in the nucleus accumbens was observed in rats with inadequate accumulation of brain DHA during development, depending on the magnitude of the change in DHA [181–183]. Thus, the effects of modulation of brain DHA on the CNS dopamine systems appear to be more dependent on the specific manipulation than the other systems discussed here.

### 7.5. Effects on Neuroinflammation

Neuroinflammation is becoming increasingly recognized as another likely contributor to the underlying pathology of depression. Of note, higher circulating levels of several NF*κ*B-regulated inflammatory mediators including interleukin-1*β*, interleukin-6, tumor necrosis factor-*α*, and interferon-*γ* have been noted in depressed patients [184–186]. Depressed patients also exhibited augmented NF*κ*B and interleukin-6 responses to psychological stressors [187]. Postmortem studies of brain from patients with major depression, or who completed suicide, also indicated increased levels of transmembrane tumor necrosis factor-*α* in some cortical regions, as well as increased expression of genes involved in inflammatory responses [188–191]. Furthermore, studies in postpartum women indicated increased levels of inflammatory mediators in those with depressive symptoms or who had previously suffered from major depression [192–194].

N-3 PUFAs have a variety of anti-inflammatory activities [195]. DHA is the precursor of neuroprotectin D1, a mediator formed in brain that inhibits the production of tumor necrosis factor-*α* and interferon-*γ* by activated T cells [25, 196]. DHA and EPA are also precursors of a variety of resolvins which control the magnitude and duration of the inflammatory response [197]. In addition, DHA and EPA inhibit the NF*κ*B-mediated inflammation cascade through actions at the toll-like 4 receptor and PPARs [198, 199]. Consistent with these activities, treatment with either DHA or EPA reduced expression of a number of inflammatory mediators including tumor necrosis factor-*α*, interleukin-6, nitric oxide synthase, and cyclooxygenase 2, and induced expression of heme oxygenase-1 in cultured BV-2 microglia [200], indicating potential anti-inflammatory mechanisms through which n-3 PUFAs could exert antidepressant effects. However, the interaction of low tissue and/or dietary n-3 PUFAs with the postpartum state has not been investigated.

### 7.6. Effects on Depression-Related Behavior

Although there is debate regarding the extent to which subhuman species can experience depression [201], several rodent models have been proven highly reliable as drug screens for the prediction of antidepressant efficacy. Among these tests, the forced swim test is perhaps the most validated [202] and is sometimes also used as a putative rodent model of depression. In the test, rats placed in an inescapable cylindrical tank of cool water are evaluated for time spent climbing, swimming, floating immobile, and, in some studies, latency to immobility. Drugs that decrease the time spent floating immobile, or increase the latency to immobility, are likely to have antidepressant effects in humans [202, 203].

In the forced swim test, postpartum rats with decreased brain DHA content exhibited shorter latencies to immobility than postpartum rats with normal brain DHA levels [49]. This effect was not observed in virgin females with decreased brain DHA indicating that it represents an interaction of the decrease in brain DHA content with the postpartum state. Shorter latency to immobility is also consistent with an interpretation of a more “depressed” phenotype in the postpartum rats with decreased brain DHA to the extent possible within the limitations of the test.

Concordant with these findings, manipulation of n-3 PUFAs in other rodent models also point to a role for lower dietary and tissue n-3 PUFA status contributing to “depressed” behavior in antidepressant drug screens. For example, adult rats fed an n-3 PUFA-deficient diet beginning at weaning, which resulted in brain DHA levels 36% lower than controls, exhibited more time immobile in the forced swim test [204]. Conversely, rats or mice that were fed n-3 PUFA-supplemented diets exhibited less immobility [132, 205–207]. Likewise, adult male mice that were treated with injections of *α*-linoleic acid also exhibited less immobility [131]. Similar effects have also been reported in the tail suspension test, another rodent antidepressant drug screen [131, 132].

## 8. Conclusion

Although a confluence of genetic and environmental factors may be required to cause depression, an individual factor (e.g., reduced brain DHA content), may create a state of vulnerability that contributes to the development of the disease when the other appropriate factors are present. The preponderance of the literature indicates that changes in brain LC-PUFA status, particularly decreased DHA, are associated with both non-puerperal and postpartum depression. Furthermore, experimentally induced reductions in brain DHA content result in neurobiological alterations in rats similar to those observed in depressed humans. These effects of decreased brain DHA interact with the postpartum state such that the number of neurobiological alterations in postpartum rats with decreased brain DHA is greater than in virgin females with decreased brain DHA, and the magnitude of some of the alterations appears to be greater in the postpartum state. With the low n-3 PUFA content of the North American diet, there is considerable potential for individuals to have suboptimal availability of these fatty acids. Genetic polymorphisms that confer suboptimal metabolism or utilization of LC-PUFA, or the physiological demands of pregnancy and lactation, may place certain individuals at even greater risk. Accordingly, decreased brain DHA, and perhaps other n-3 PUFAs, represents an important potential risk factor for depression generally, and postpartum depression in particular. 

Despite this growing body of evidence, the role(s) of LC-PUFA in the pathogenesis of postpartum depression and other depressive illnesses remains to be fully elucidated. In addition to determining specifically how changes in brain LC-PUFA composition contribute to the etiology of depression (e.g., altered membrane properties, actions of LC-PUFA-derived mediators, etc.), it must be determined whether n-3 PUFA status contributes to the etiology of depression in all, or only a subset of, patients (e.g., postpartum females). Importantly, the reversibility of the neurobiological consequences of a pregnancy-associated loss of brain DHA must be determined. Should these changes prove to be reversible, this will support the use of n-3 PUFA supplements in the treatment of postpartum depression. On the other hand, should the neurobiological consequences of a pregnancy-associated loss of brain DHA be irreversible, this will indicate the imperative of preventing the loss of DHA during pregnancy and lactation through appropriate nutrition and/or supplementation. Finally, should such findings support the viability of preventing postpartum depression and/or treating existing depressive illness with n-3 LC-PUFAs, the appropriate formulation, optimal dose, and treatment duration also remain to be determined in well-designed, adequately powered clinical trials.

## Figures and Tables

**Table 1:** Double-blind, randomized, placebo-controlled trials of n-3 PUFAs in nonpuerperal depression.

Author	Year	Disorder	Population	Intervention	Treatment groups^1^	Dose	Duration	Major finding
Nemets et al. [94]	2002	Major depressive disorder	Israel, 85% female	Add-on to current antidepressant	Ethyl-EPA, *n* = 10 placebo (not stated), *n* = 10	2 g/day	4 weeks	Improvement in HDRS score over placebo (*P* < .05)
Peet and Horrobin [95]	2002	Major depressive disorder	UK, 84% female	Add-on to current antidepressant	ethyl-EPA 1 g/kg, *n* = 17 2 g/kg, *n* = 18 4 g/kg, *n* = 17 placebo (liquid paraffin), *n* = 14	1, 2, or 4 g/day	12 weeks	Improvement in HDRS score over placebo at 1 mg/kg (*P* < .05). No effect at 2 or 4 mg/kg
Su et al. [96]	2003	Major depressive disorder	Taiwan, 82% female	Add-on to current antidepressat	Fish oil, *n* = 12, placebo (olive oil ethyl esters), *n* = 10	9.6 g/day containing EPA: 4.4 g/day DHA: 2.2 g/day	8 weeks	Improvement in HDRS score over placebo (*P* < .05)
Marangell et al. [104]	2003	Major depressive disorder	USA, 80% female	Monotherapy	DHA, *n* = 18 placebo (not stated), *n* = 7	2 g/day	6 weeks	No effect of treatment on HDRS, CGI or MADRS scores
Silvers et al. [106]	2005	Depression	New Zealand, 53% female	Add-on to current antidepressant	Fish oil, *n* = 40 placebo (olive oil), *n* = 37	8 g/day containing EPA: 0.6 g/day DHA: 2.4 g/day	12 weeks	Improvements in HDRS and BDI scores were greater than, but not significantly different from placebo
Nemets et al. [97]	2006	Childhood depression	Israel, Gender ratio not stated	Monotherapy	EPA + DPA, *n* = 10 placebo (olive or safflower oil), *n* = 10	1 g/day containing EPA: 400 mg/day DHA: 200 mg/day	16 weeks	Improvement in CDRS score over placebo (*P* < .05)
Grenyer et al. [108]	2007	Major depressive disorder	Australia, 74% female	Add-on to current antidepressant (74% of subjects) or Monotherapy	Fish oil, *n* = 32 placebo (olive oil), *n* = 28	8 g/day containing EPA: 0.6 g/day DHA: 2.2 g/day	16 weeks	No effect of treatment on BDI score
Su et al. [98]	2008	Major depression during pregnancy	Taiwan, 100% female	Monotherapy	EPA+DHA, *n* = 13 placebo (olive oil ethyl esters), *n* = 11	EPA: 2.2 g/day DHA: 1.2 g/day	8 weeks	Improvement in HDRS over placebo (*P* < .05) and higher response rate (*P* < .05)
da Silva et al. [101]	2008	Depression in Parkinson's disease	Brazil, Gender ratio not stated	Monotherapy or add-on to current antidepressant	Fish oil only, *n* = 6, Fish oil + antidepressant, *n* = 8, placebo (mineral oil), *n* = 7, placebo + antidepressant, *n* = 8	EPA: 720 mg/day DHA: 480 mg/day	12 weeks	Improvement in MADRS score compared to placebo or placebo + antidepressant (*P* < .05)
Rogers et al. [105]	2008	Mild to moderate depression	UK, Gender ratio not stated	Monotherapy	EPA + DHA, *n* = 96, placebo (olive oil with 7.5 mg mixed tocopherols), *n* = 94	EPA: 630 mg/day, DHA: 850 mg/day	12 weeks	No effect of treatment on DASS or BDI scores
Lucas et al. [99]	2009	Middle-aged women with psycholo gical distress and depressive symptoms	Canada, 100% female	Monotherapy	Ethyl-EPA, *n* = 59 placebo (sunflower oil), *n* = 61	1.5 g/day containing EPA: 1.05 g/day DHA: 0.15 g/day	8 weeks	Improvement in HDRS score was greater than placebo only for subjects not meeting criteria for major depression (*P* < .05)
Mischoulon et al. [107]	2009	Major depressive disorder	USA, 65% female	Monotherapy	Ethyl-EPA, *n* = 11 placebo (paraffin oil with 0.2% *α*-tocopherol), *n* = 3	1 g/day	8 weeks	Improvement in HDRS score was greater than, but not significantly different from placebo
Rondanelli et al. [100]	2010	Elderly women with depression	Italy, 100% female	Monotherapy	EPA + DHA, *n* = 22, placebo (paraffin oil), *n* = 24	EPA: 1.67 g/day, DHA: 0.83 g/day	8 weeks	Improvement in GDS score over placebo (*P* < .05).
Bot et al. [109]	2010	Major depression in diabetes mellitus	The Netherlands, 52% female	Add-on to current antidepressant	Ethyl-EPA, *n* = 12 placebo (rapeseed oil with medium chain triglycerides), *n* = 12	1 g/day	12 weeks	No effect of treatment on MADRS score

^1^Sample size at end of study.

HDRS:Hamilton Depression Rating Scale, CGI:Clinical Global Impression, MADRS:Montgomery-Åsberg Depression Rating Scale, BDI:Beck Depression Inventory, CDRS:Childhood Depression Rating Scale, DASS:Depression, Anxiety, and Stress Scales, GDS:Geriatric Depression Scale.
